# *In situ* Enabling Approaches for Tissue Regeneration: Current Challenges and New Developments

**DOI:** 10.3389/fbioe.2020.00085

**Published:** 2020-02-18

**Authors:** Juliana R. Dias, Nilza Ribeiro, Sara Baptista-Silva, Ana Rita Costa-Pinto, Nuno Alves, Ana L. Oliveira

**Affiliations:** ^1^Centre for Rapid and Sustainable Product Development, Polytechnic Institute of Leiria, Leiria, Portugal; ^2^CBQF – Centro de Biotecnologia e Química Fina, Laboratório Associado, Escola Superior de Biotecnologia, Universidade Católica Portuguesa1, Porto, Portugal

**Keywords:** *in situ* approaches, tissue regeneration, bioprinting, *in situ* biomaterials, computer/non-computer assisted approaches

## Abstract

*In situ* tissue regeneration can be defined as the implantation of tissue-specific biomaterials (by itself or in combination with cells and/or biomolecules) at the tissue defect, taking advantage of the surrounding microenvironment as a natural bioreactor. Up to now, the structures used were based on particles or gels. However, with the technological progress, the materials’ manipulation and processing has become possible, mimicking the damaged tissue directly at the defect site. This paper presents a comprehensive review of current and advanced *in situ* strategies for tissue regeneration. Recent advances to put in practice the *in situ* regeneration concept have been mainly focused on bioinks and bioprinting techniques rather than the combination of different technologies to make the real *in situ* regeneration. The limitation of conventional approaches (e.g., stem cell recruitment) and their poor ability to mimic native tissue are discussed. Moreover, the way of advanced strategies such as 3D/4D bioprinting and hybrid approaches may contribute to overcome the limitations of conventional strategies are highlighted. Finally, the future trends and main research challenges of *in situ* enabling approaches are discussed considering *in vitro* and *in vivo* evidence.

## Introduction

Every year, millions of people around the world suffer from tissue damage, either due to disease, trauma, or simply aging (Badylak and Nerem, 2010; O’Brien, 2011). Although autografts and allografts are considered gold standard procedures for organ transplantation, many limitations remain, such as a donor shortage, donor site morbidity, and adverse immune responses (Atala, 2004; Dias et al., 2016). The need for alternative approaches has led to the foundation of tissue engineering (TE), an exciting field of research that over the last 25 years has used materials in the presence or absence of cells and biochemical factors to restore, maintain, or improve biological tissues (Langer and Vacanti, 1993). During this period, scientists have recognized that tissue reconstruction is an extremely complex task that cannot be performed by simply bringing cells and materials together (Hoffman et al., 2019). The exciting path of TE, however, allowed the scientific community to gain a deeper understanding of cell and stem cell biology, uncovering the importance of the properties of the extracellular matrix (ECM) in directing cell behavior. Cells are intrinsically sensitive to local signals from the macro- to the nanoscale, including both chemical (e.g., a specific molecule recognition) and physical (e.g., topographical pattern) signals (Crowder et al., 2016). In addition, they can be stimulated by the mechanical environment (tissue-like stiffness) (Chowdhury et al., 2010) or when exposed to shear fluid rates close to that of the target biological system (Blaeser et al., 2016).

As our understanding of how the physical and chemical properties of the ECM direct stem cell fate and tissue formation has evolved, we have observed a simultaneous development of advanced biomaterials with the capacity to recapitulate such properties locally at the tissue interface (Stuart et al., 2010; Dhowre et al., 2015). These smart materials can be dynamically altered by chemistry, enzymes, light, and mechanics, among others (Momeni et al., 2017; Miri et al., 2018).

To implant tissue-specific biomaterials alone or in combination with cells and biomolecules at the site of the tissue defect is a concept that has been defined as *in situ* tissue regeneration. This approach takes advantage of the surrounding biological microenvironment, directing the fate of cells to regenerate new tissue without complicated prior *in vitro* cell manipulation (Li et al., 2015). Several reviews can be found in the literature describing what is currently known about the mechanisms and strategies used to induce *in situ* cell differentiation and tissue regeneration (Chen et al., 2011; Yang et al., 2014; Talacua et al., 2015; Dhand et al., 2016; Murdock and Stephen, 2017; Wissing et al., 2017).

A very important aim for *in situ* tissue regeneration strategy is the development of technologies to manipulate and process materials at the defect site, recreating the tissue’s size and shape in the surgical room. This implies that the selection of materials must meet new requirements, while the processing technologies need to be more sophisticated to meet the demands of portability, adaptability, and simplicity for use over a relatively short period of time. Therefore, *in situ* approaches need state-of-the-art customized processing technologies. The advances in micro- and nano-fabrication technologies can provide the platforms to engineer structures at high resolution with the capacity to fully recreate *in situ* the complexity of a tissue defect (Holzmond and Li, 2017; Bella et al., 2018; Hakimi et al., 2018; Hudita et al., 2019).

Progress in areas such as cell culture automation, techniques for cell sorting, and new material formulations for biofabrication, have generated more efficient therapies for preclinical models as well as to repair simple tissues in the laboratory. Although self-sustaining solutions that facilitate full tissue integration and homeostasis in a timely manner remain elusive, there has been great progress in the development of technological strategies for *in situ* tissue regeneration.

This review provides a perspective on the potential and state of development of different *in situ* fabrication technologies for multiple tissue regeneration, such as non-computer-based approaches (*in situ* spraying and spinning, and *in situ* hydrogel-based strategies) and computer-based approaches (*in situ* 3D/4D bioprinting and *in situ* hybrid approaches). The next generation of constructs should, ideally, not only mimic the organs or tissues’ architecture and properties but also consider the dynamics of material and the cell-material interaction.

## Materials for *in situ* Tissue Regeneration

**FIGURE 1:**
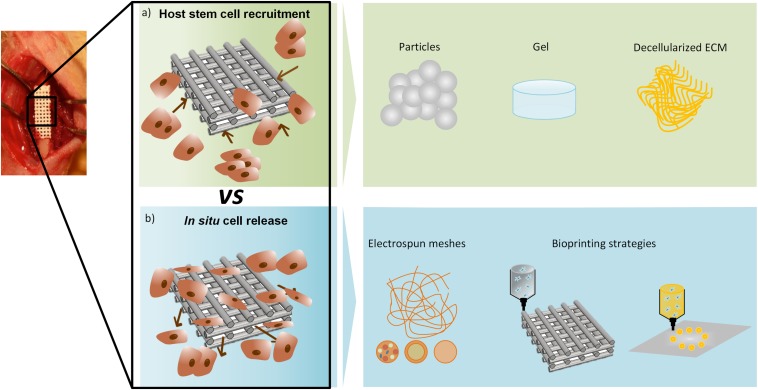
*In situ* regeneration approaches. **(a)** Host stem cell recruitment- biostructures (particles, gel or decellularized ECM) recruit cells from the surrounding environment; **(b)** *In situ* cell release – cells are released from the biostructure to the surrounding environment, commonly produced by electrospinning or bioprinting strategies.

Initially, *in situ* tissue regeneration was proposed mainly as a strategy to avoid complicated and long-term *in vitro* cell isolation, expansion, and maturation (Li et al., 2015). Additionally, this approach overcomes the poor integration of *in vitro* new tissue that commonly occurs after implantation (Smits et al., 2016). Based on this, *in situ* tissue regeneration intends to promote regeneration, taking advantage of the native microenvironment of the injury site through the implantation of tissue-specific biomaterials combined or not with biomolecules or cells (Figure 1) (Li et al., 2015; Zhao et al., 2017). This approach is a dynamic process in which cells recruited from host or delivered by the scaffold must proliferate, self-assemble, and differentiate before reaching a steady-state (Murdock and Stephen, 2017). The *in situ* tissue regeneration takes advantage of native biochemical and biophysical cues that, contrary to what happens in a bioreactor, is a simple, scalable, and cost-effective methodology (Murdock and Stephen, 2017).

Regardless of the *in situ* fabrication strategy used, biomaterials have a key role in the regeneration process. Initially, *in situ* regeneration approaches focused on cell recruitment to the injury site through biomaterials and/or biological cues (Ko et al., 2013; Lee et al., 2016). However, it is well known that a non-target-specific scaffolding system lacks with the formation functional tissue (Sengupta et al., 2014). Consequently, the best approach to achieve an appropriate tissue regeneration comprises the combination of scaffolds with cells or biomolecules that mimic the injury target tissue/organ with immunomodulatory properties (Lee et al., 2016; Smits et al., 2016). Natural and synthetic materials are suitable for *in situ* regeneration if they are biodegradable, have a minimal pro-inflammatory response, have anti-inflammatory properties, and promote an immunomodulatory response (Li et al., 2015; Murdock and Stephen, 2017). Additionally, the physical properties, chemical composition, and biological functions of the material must modulate the cellular response (adhesion, proliferation, and differentiation) as well as neo-tissue formation (Li et al., 2015). Commonly, *in situ* biomaterials are categorized as natural biomaterials, synthetic polymers, bioceramics, and ECM-based materials (Li et al., 2015; Murdock and Stephen, 2017). Natural biomaterials including polysaccharides (such as cellulose, alginate, hyaluronic acid, starch, dextran, heparin, chitin, and chitosan) and proteins (collagen, gelatin, and fibrin) have been broadly used in *in situ* regeneration due to their ECM similarity and recognition sites (Li et al., 2015; Lee et al., 2016). Contrary to natural biomaterials, synthetic polymers present good mechanical properties and can be manufactured easily with high precision (Li et al., 2015; Murdock and Stephen, 2017). Commonly used synthetic polymers *in situ* are poly(L-lactic acid) (PLLA), polyurethane (PU), poly(lactic-co-glycol acid) (PLGA), and poly(caprolactone) (PCL), although the lack of functional groups limits their cell affinity (Sengupta et al., 2014; Murdock and Stephen, 2017). Bioceramics, of natural or synthetic origin, are characterized by their biocompatibility, osteoconductivity, corrosion resistance, and a hard and brittle structure (Pina et al., 2017). Depending on the type of ceramics used, their interaction with the host tissue can be categorized as bioinert or bioactive, being the latest resorbable or non-resorbable (Vallet-Regí and Ruiz-Hernández, 2011). Bioceramics can be used as powders, granulates, or coatings, as well as being processed by additive manufacturing techniques, making it possible for them to mimic the architecture of bone ECM (Pina et al., 2017; Ma et al., 2018). The fourth category, ECM-based materials, is one of the most promising strategies due to the provided microenvironment which mimics the native ECM (Li et al., 2015; Murdock and Stephen, 2017). Despite ECM-based materials can be derived from decellularized tissues that are similar to the native tissue present as major limitation the donor shortage (Crapo et al., 2011; Li et al., 2015). Nevertheless, due to the technological advances, it is possible to create an ECM-based materials that mimic the native tissues in terms of its architecture and biomechanical properties and allows cells and biomolecules to be incorporated (Li et al., 2015; Lee et al., 2016). It is important to highlight that in most of the *in situ* approaches the use of biomaterials is required (regardless of their shape or size), either being by injection, surgery, or insertion into the defect. This will injure the tissues or organs involved. Consequently, the use of minimally invasive and time-effective approaches are required.

## *In situ* Enabling Approaches

The TE field has been growing exponentially during the past few years, exploring the regeneration of several tissues (e.g., bone, skin, ligaments, tendons, and cartilage, among others) using different approaches, namely top-down (cells are seeded onto a prefabricated porous scaffold) or bottom-up (scaffolds are fabricated combining modular units such as biomaterials, cells, growth factors and biomolecules) (Dias et al., 2016). Presently, the manipulation and processing of materials in the surgical room takes materials science to a new level of sophistication, but at the same time, highlights the need for easy application processes, using technologies that could be as much as possible minimally invasive, intuit and easy to manipulate for the surgeons, in order to meet the time constrains of a clinical interventions, while maintaining all the safety and sterility requirements needed. This implies that the selection of materials must meet new specifications, while the processing technologies need to be more refined to meet the demands of portability, adaptability, and simplicity for use over a relatively short period of time. The *in situ* enabling technologies can be categorized in non-computer-assisted (defect area is fulfill with non-defined deposition pattern) or computer-assisted approaches (the defect is scanned and deposition pattern defined accordingly).

### Non-computer-Assisted Approaches

#### *In situ* Spinning and Spraying

*In situ* spinning and spraying are technologies that are now giving their first steps toward a clinical application. Both approaches intend to deposit fibers or particles into the defect area in a non-predefined strategy. Electrospinning has attracted great interest in TE applications, by generating a diversity of nanoscale fibers ranging from a few microns to less than 100 nm, made of polymers, ceramics, or their composite scaffolds (Teo and Ramakkrishna, 2006; Bhardwaj and Kundu, 2010; Shi et al., 2010; Yang et al., 2018). In this technique, a polymer solution or melt is added to a capillary and a small droplet (a Taylor cone) is formed at the tip of the needle due to the extrusion of the precursor spinning solution by the syringe pump in the presence of a high electric voltage (Yarin et al., 2001). When the force between the Taylor cone and the grounded collector under an electric field is between 5 and 30 kV, it is able to overcome the solution surface tension and a thin jet of the charged solution is accelerated toward the target collector. If the solution is viscous enough to stabilize the jet, the polymer solution is severely stretched and the solvent evaporates to form ultrathin fibers that solidify and deposit on the collector forming a non-woven mesh of fibers (Greiner and Wendorff, 2007). However, when the collector speed is increased, the fibers are rapidly taken up on the surface of the collector tightly in a circumferential manner, resulting in a high alignment. The final fiber diameter depends on various parameters, including the applied voltage, the distance between the tip and the collector, the solvent type, the solution feed rate, the needle diameter, and the concentration of the polymer solution (Matthews et al., 2002; Li and Xia, 2004; Inai et al., 2005; Beachley and Wen, 2009; Liao et al., 2012).

These nano-sized fibers that mimic the native ECM have been successfully electrospun, including biodegradable, non-degradable, natural, and synthetic polymers such as collagen, silk, gelatin, elastin, alginate, chitosan, PGA, PLLA, PLGA, and PCL (Matthews et al., 2002; Huang et al., 2003; Min et al., 2004; Yang et al., 2005; Buttafoco et al., 2006; Choi et al., 2008; Klossner et al., 2008; Zeugolis et al., 2008; Hartman et al., 2009; Chen et al., 2010; Liu et al., 2010; Zhou et al., 2010; Bonino et al., 2011). Furthermore, the standard electrospinning setup can be modified by replacing a single spinneret (nozzle) with a coaxial nozzle to produce a single polymeric fiber composed of two different simultaneously spun polymers, the structure of which consist of a core-shell assembly (Loscertales et al., 2004; Wang et al., 2006). Another approach is dual nozzle electrospinning that enables the combination of a variety of polymers prepared in independent solvents and electrospun at the same time to make a hybrid scaffold. Additionally, the use of different collectors allows control of the deposited fiber orientation (Teo and Ramakkrishna, 2006). This further highlights the versatility, reproducibility, scalability, and sustainability of the electrospinning technique to produce different nanofibrous structures with relevant potential for biomedical applications.

**FIGURE 2:**
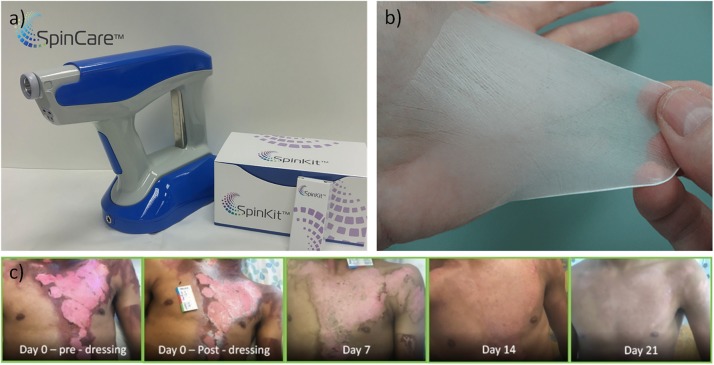
**(a)** Handheld electrospinning device marketed by Nanomedic Technologies Ltd. Israel, **(b)** electrospun fiber deposition, **(c)** clinical case report, treatment of second degree burn, modified from Nanomedic Technologies Ltd (2019).

The incorporation of electrospinning in *in situ* biomedical applications creates important challenges for how to re-design conventional electrospinning devices, which use high voltages and have limited portability, to become portable user-friendly equipment that can be used in the “patient’s bed.” Therefore, great efforts have been made to develop new portable electrospinning devices for the purposes of wound dressing and regeneration (Xu et al., 2015; Haik et al., 2017). An example is the SkinCare (Figure 2), a portable wound care system developed by Nanomedic Technologies Ltd (2019) in Israel. Yan et al. (2019) reflects precisely the recent advances in portable electrospinning technology for the *in situ* delivery of personalized wound care and regenerative medicine. The currents involved in the electrospinning process are in the order of nanoamperes. This has inspired pioneering work on cell-spinning, where post-electrospinning cells were shown to remain viable (Jayasinghe, 2013). Indeed, the implementation of spinning techniques, together with the use of living cells in order to mimic the complexity of the native tissues, might lead to the development of functional biomaterials compatible with *in situ* TE and subsequent successful clinical outcomes (Poncelet et al., 2012; Chen et al., 2015).

As an alternative to *in situ* electrospinning, there are a few studies that have reported a modified spinning technique, namely, air flow-directed *in situ* electrospinning, which can precisely control the deposition of polymeric fibers on a desired site under the assistance of air flow (Jiang et al., 2014; Dong et al., 2015; Lv et al., 2016). For example, Lv et al. (2016) applied this technology to precisely deposit medical glue based on octyl-2-cyanoacrylate (NOCA) ultrathin fibers onto dura defects in goat brains to improve its sealing capability, to avoid tissue adhesion, and to save time required for conventional dura suture. In another work, the airflow assisted *in situ* precision e-spinning was compared to the traditional spraying method in the delivery of NOCA adhesive fibers by using a model of liver injury in rats. Compared with spraying, *in situ* precision e-spinning improved operational performance and safety by precisely depositing a sufficiently small amount of cyanoacrylate fibers onto a wound, in addition to producing a rapid and complete hemostatic effect (e.g., minor hepatocyte injury, moderate inflammation, and a significant ability for liver regeneration) (Dong et al., 2015).

Electrospraying (ES) or electrohydrodynamic atomization consists of a jetting methodology that relies on a potential difference between two electrodes for accelerating a column of liquid (Jaworek, 2007). The basis set up of ES is quite similar to that used in the electrospinning technique; briefly consists of a high voltage supply, a syringe pump, a syringe with a metallic needle and a collector. In ES, when the electrical field is applied the liquid jet is broken into fine liquid droplets with similar charge promoting their dispersion (Jaworek, 2007). The electric charge creates an electrostatic force inside the droplet which is able to overcome its surface tension (Boda et al., 2018). Subsequently, this excess of charge will be dissipated and smaller charged droplets on the micro to nanoscale will be ejected toward the collector (Jaworek and Sobczyk, 2008). These electrosprayed droplets are commonly unstable and evaporate, generating micro- and nanoparticulates (Bock et al., 2012).

The temperature and humidity affect the vaporization of the solvent from the droplets generated during ES. Generally, the particles are collected in ethanol or a water-ethanol mixture or other water-based systems, since most of the polymers are insoluble in water (Boda et al., 2018). Consequently, organic solvents with high vapor pressure or a low surface tension are the most suitable for ES (Jaworek and Sobczyk, 2008). Commonly, it is easier to electrospray high molecular weight (Mw) polymers at low solute concentrations in organic solvents than low Mw polymers (Boda et al., 2018).

Several types of natural polymers, such as chitosan (Songsurang et al., 2011), alginate (Park et al., 2012), cellulose (Huang et al., 2012), or collagen (Nagarajan et al., 2014), as well as synthetic PLGA (Nath et al., 2013), PCL (Hwang et al., 2008), and polystyrene (PS) (Zheng et al., 2006) have been successfully electrosprayed to generate nano/micro-particulate systems.

One of the potential applications of electrospraying is bioelectrospraying (BES), which consists of the *in situ* delivery of living cells (Braghirolli et al., 2013) or encapsulated cells (Minglin et al., 2013) for TE and regenerative medicine (TERM) applications (Jayasinghe, 2011). The use of bioelectrospraying to process cell suspensions into 3D architectures for generating functional tissue constructs has shown promising results in the past few years (Jayasinghe, 2011).

Rabbit bone marrow stromal cells (BMSCs) subjected to electrospraying with 6 mL/h of flow rate of and voltage of 7.5–15 kV did not show deleterious effects on their morphology, proliferation, or multilineage differentiation potential (Sambit et al., 2010). Human deciduous tooth pulp MSCs exposed between 15 and 60 min to 15 kV voltage, with a 0.46 mL/h flow rate, did not present a significant decrease in cell viability, proliferation, plasticity, or immunophenotypic profile, although some DNA damage was observed after 30 min of BES, which could be self-repaired after 5 h of culture (Braghirolli et al., 2013). Abeyewickreme et al. (2009) described that bioelectrosprayed mouse embryonic stem cells (ESCs) did not show alterations in their pluripotency.

**FIGURE 3:**
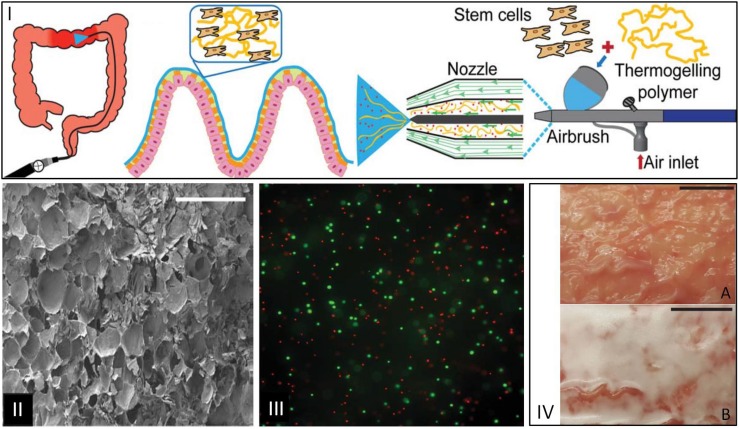
**(I)** Illustration of therapeutic delivery method of a cellular hydrogel coating on intestinal tissue via a spray device. **(II)** SEM images of lateral cross sections of hydrogels with TGM:PAMAM (1:1), scale bar: 500 mm. **(III)** Fluorescence microscopy image of live(green)/dead(red)-treated sections of 10 wt% TGM and 10 wt% PAMAM hydrogels sprayed at 0.5 bar with dual-stream method. **(IV)** Images of (A) porcine intestinal tissue and (B) hydrogel formation on tissue after spraying for 1 min, scale bar: 1 cm (Pehlivaner et al., 2016).

Pancreatic islets encapsulated in an alginate shell-matrigel core configuration were processed by coaxial ES and were able to maintain *in vitro* and *in vivo* viability and glucose regulating functionality after transplantation in immune competent mice (Minglin et al., 2013). In another study, alginate microcapsules with core-shell structures plus rat pancreatic islets were produced. When implanted in a type I diabetic mouse model, it was shown that these hydrogel microcapsules with core-shells improved islet cells encapsulation and conferred immune protection (Zhao et al., 2014). A novel approach combining microfluidic cell encapsulation with electrospinning based membranes was developed using a layer-by-layer process by alternating fiber electrospinning and cell spraying (Weidenbacher et al., 2017). This system conferred a temporary protection for the cells delivery (C2C12 cell line) to produce a fibrous cell-laden hybrid biograft (Weidenbacher et al., 2017). *In situ* thermoresponsive hydrogels composed of thermogelling macromers (TGM) of Poly(N-isopropylacrylamide) (pNiPAAm) sprayed with a high polyamidoamine (PAMAM) concentration at low pressure with encapsulated cells were deposited using single-stream, layer-by-layer, and dual-stream spraying as coatings on interior intestinal porcine tissue, to create a homogeneous therapeutic coating on the area to further treat lesions triggered by inflammatory bowel disease (Figure 3) (Pehlivaner et al., 2016).

Coaxial ES was used to encapsulate murine ESCs into alginate microcapsules, and showed a higher pluripotency of the ESCs according to their differentiation into cardiomyocytes and higher expression of cardiomyocyte specific gene markers than the cells cultured on 2D substrate or simple 3D alginate microbeads (Zhao et al., 2014).

Human osteoarthritic chondrocytes and chondrons (choncrocytes with their pericellular matrix) in fibrin gel were airbrush sprayed using a commercial device (Baxter) and cultured for 21 days to assess matrix production (De Windt et al., 2015). To investigate the feasibility of the sprayed cell-based fibrin glue scaffold, a full-thickness sized cartilage defect was created arthroscopically in a cadaver model, and the defects were filled with either arthroscopic airbrushing or needle extrusion. Results showed that both chondrons and chondrocytes can be distributed in a sprayed fibrin glue scaffold without affect the cell viability and supporting the matrix production (De Windt et al., 2015).

Platelet origin fibrin gel was used for spray painting *in situ* to create a patch in a mouse model of myocardial infarction (Tang et al., 2017). Platelet rich plasma and calcium-containing media solution were sprayed in two different syringes with compressed CO_2_ onto the heart of mice to produce a uniform patch, which was found to promote cardiac repair and reduce cardiac dysfunction after myocardial infarction (Tang et al., 2017).

Spray seeding is a technology that can be used for the delivery of progenitor cells, Schwartz et al. (2017) have studied the impact of spraying as a seeding strategy for repopulation of decellularized small intestine onto decellularized scaffolds. In this work a new spray device (3D Spray) has been used to seed intestinal organoids to form epithelium on decellularized intestinal ECM scaffolds as *in vitro* models (Schwartz et al., 2017).

Electrospray technology is one of the most promising approaches in the TERM field, considering its wide availability, easiness to set up and operate, reusability, and high production rate. *In situ* electrospraying using cells with or without polymers – bioelectrospraying – is of ultimate importance given the need to create a fully 3D tissue, a concept that will easily be translated to the clinic. However, compared to the *in situ* bioprinting, present as the main limitations the random deposition and the poor ability to fulfill a 3D defect.

Furthermore, *in situ* spinning and spraying allow for the creation of structures either fibrilar or composed by agregated micro/nano particles, which have typically small porosity and are not ideal to build large, highly porous 3D constructs. These are line-of-sight technologies that project the material and cells onto the defect rather than building a precise shaped geometry as in case of 3D printing. Therefore, it is then clear that skin wound healing is the most important targeted application for these technologies. The gold standard are portable devices that are directly manipulated by the clinicians in a simple manner, controlling the amount of materials/cell to be added, the most adequate angle for projection or the moment when the defect is fulfilled. This means that in most of the cases it is not necessary to scan the damage area to have a defined computerized shape of the defect. Nevertheless its existence can help to predict the extension of the lesion and evaluate the exact amount of material/cells to be used.

To find the adequate polymers to bring adequate structure integrity, able to promote tissue regeneration and to guarantee that the *in situ* process does not affect the surrounding cells and tissue integrity are still challenges to overcome.

#### *In situ* Gelling

**FIGURE 4:**
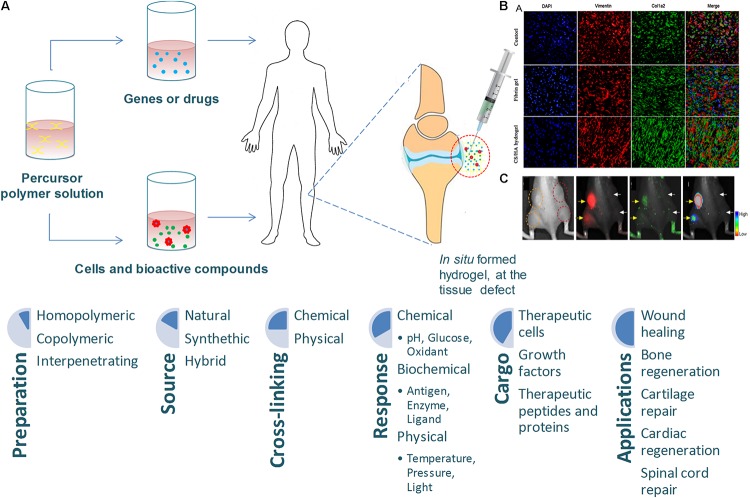
**(A)** Injectable hydrogels for tissue engineering applications, adapted from Choi et al. (2015). **(B)** Representative immunofluorescent images of collagen deposition, adapted from Deng et al. (2017). **(C)** *In vitro* data of sericin hydrogels injected subcutaneously, adapted from Wang et al. (2014).

Hydrogels are 3D polymeric networks with a wide potential in the field of TE due to their ability to support cell proliferation, migration and differentiation, allow oxygen and nutrient transport, and mimic native tissues. The importance of these biomaterials in the field of TE when applied *in situ* have been recently highlighted (Park and Park, 2018). These networks might be composed of crosslinked natural polymers (e.g., alginate, chitosan, gelatin, silk) or synthetic macromolecules [e.g., PEG, polyvinyl alcohol (PVA)] (Marchesan et al., 2013; Lee and Kim, 2018). These highly hydrated networks can be stabilized via physical or chemical crosslink (Moura et al., 2013). Hydrogels might also be biodegradable, responsive to specific stimuli (i.e., pH or temperature) (Toh and Loh, 2014; Dabiri and Damstetter, 2016; Ribeiro et al., 2018), and engineered to deliver therapeutic compounds in a sustained and controlled released way, exhibiting properties such as adhesiveness, elasticity, and biocompatibility (Toh and Loh, 2014). These matrices are versatile and can be adapted, making them useful for filling deep defects or those with irregular contours. On the other hand, they can be easily loaded with bioactive agents which can trigger regenerative processes at multiple cellular levels (Agubata et al., 2016). However, the success of hydrogel applications as a delivery system in TE will largely depend on the biomimetic design and engineering, and cell-material interactions involved in cell fate (Figure 4) (Toh and Loh, 2014; Dabiri and Damstetter, 2016; Nie et al., 2016).

Recent findings underscore the need for the development and implementation of novel *in situ* hydrogel driven therapies. The most pioneered advances in *in situ* hydrogel technology have contributed to a paradigm shift in wound healing applications. One of the most notable examples is an injectable gelatin microcryogel, which can load cells for enhanced cell delivery and cell therapy for wound healing (Zeng et al., 2015). In Le Thi et al. (2017) study, human adipose-derived stem cells (hASCs) laden in gelatin microcryogels were used as primed injectable 3D micro-niches for a new cell delivery methodology for skin wound healing. The study showed an improvement in wound bed recovery and a direct effect on the wound basal layer resulting in enhanced healing. A different gelatin hydrogel was prepared by double enzymatic crosslinks, where horseradish peroxidase (HRP) and tyrosinase were used as crosslinking agents in order to develop a novel *in situ* tissue-adhesive system (Le Thi et al., 2017). A biodegradable *in situ* thermosensitive hydrogel has been developed by Gong et al. (2013) as a controlled drug delivery system composed of a one-step solid dispersion method with curcumin and PEG-PCL copolymer for cutaneous wound repair, and has been shown to be successful. Moreover, injectable *in situ* crosslinking chitosan-hyaluronic acid based hydrogels have shown notable potential in abdominal tissue regeneration (Deng et al., 2017). Other advances have been made in spinal degenerative disease, for example an *in situ* injectable chemically crosslinked hydrogel formed by a two-component reaction of liquid silk fibroin with liquid polyurethane at physiological temperature conditions which was shown to fulfill an entire defect, reducing the danger of implant relocation and the subsequent loss of disk height, minimizing the operative trauma (Hu et al., 2017). Efforts were also made in cartilage regeneration using an injectable hydrogel with transforming growth factor (TGF)-β1, which was considered capable of promoting chondrogenesis (Zhou et al., 2017). This study suggests that the new hydrogel possesses advantages for clinical application and should further be studied for its future use in cartilage TE.

An N-isopropylacrylamide/gelatin microparticle composite hydrogel used in bone TE enhanced the mineralization within the bone defect, which significantly increased tissue infiltration and osteoid formation, suggesting that the hydrogel system facilitates bone ingrowth and integration (Vo et al., 2016). Another nanohydroxyapatite/glycol chitosan/hyaluronic acid composite hydrogel was also designed for bone TE (Huang et al., 2016). The good attachment and spread of the cells incorporated after 7 days of co-incubation highlighted the potential of the hydrogel for bone TE applications. In addition, an injectable hydrogel using chitin and poly(butylene succinate) loaded with fibrin nanoparticles and magnesium-doped Bioglass has been investigated by Vishnu Priya et al. (2016). The hydrogel induced osteogenic differentiation by stimulating the expression of alkaline phosphatase and osteocalcin, being a promising strategy for regenerating irregular bone defects. Another advantage of hydrogels is their use as carriers for bioactive compounds, thus improving their delivery performance. Ophthalmic *in situ* gels have been developed to prolong the precorneal contact time of ocular drugs. For example, sodium alginate and hydroxypropyl methyl cellulose have been used to develop a new *in situ* gel for diclofenac ophthalmic delivery (Subimol et al., 2013). Pluronic F-127, a triblock copolymer with a non-ionic nature, goes through *in situ* gelation due to temperature changes. In a work by Pagliarussi et al. (2006) a formulation containing this polymer was incorporated with clotrimazole-β-cyclodextrin complex to develop a vaginal *in situ* gel. This formulation increased the release time of the drug at the application site.

Cardiovascular are amongst the most important fields of application for *in situ* crosslinked hydrogels (Li et al., 2014; Radhakrishnan et al., 2014). Recently, several injectable hydrogels have emerged as a potential candidates for cardiac tissue regeneration due to improved patient compliance and facile administration via minimal invasive mode that treats complex infarction (Radhakrishnan et al., 2014). For instances, in a work of Steele et al. (2019) a catheter−injectable hyaluronic acid hydrogel is proposed utilizing a polymer–nanoparticle crosslinking mechanism. Due to the notable shear−thinning the hydrogel can be easily injected through a long, narrow, physiologically−relevant catheter and needle with hydrogel mechanics unchanged after delivery.

When comparing with other *in situ* approaches it is clear that while hydrogels can reach the entire volume of complex defects, they may lack in adequate mechanical properties to be able to successfully fill large hard tissue defects. On the other hand, careful design of the formulation should result in relatively fast forming hydrogel structures to be compatible with clinical application (Wang et al., 2014). Therefore the gelling time is a crucial parameter to be taken into consideration together with an ideal viscosity to allow for minimally invasive application. Soft tissue applications are no doubt the most important target of *in situ* hydrogel systems were there have been the greatest advances. At the same time, hydrogels have been gaining momentum with the development of computer-aided bioprinting technologies, to create more precise, porous 3D constructs, openning the range of applications and creating the possibility for loading bioactive factors or cells during fabrication in a spacially controlled manner (Hakimi et al., 2018; Albanna et al., 2019). However, the use of prefabricated hydrogel scaffolds brings complexity to the TE approach as compared to the procedure of directly inject a gel *in situ*.

### Computer-Assisted Approaches

#### 3D *in situ* Bioprinting

**FIGURE 5:**
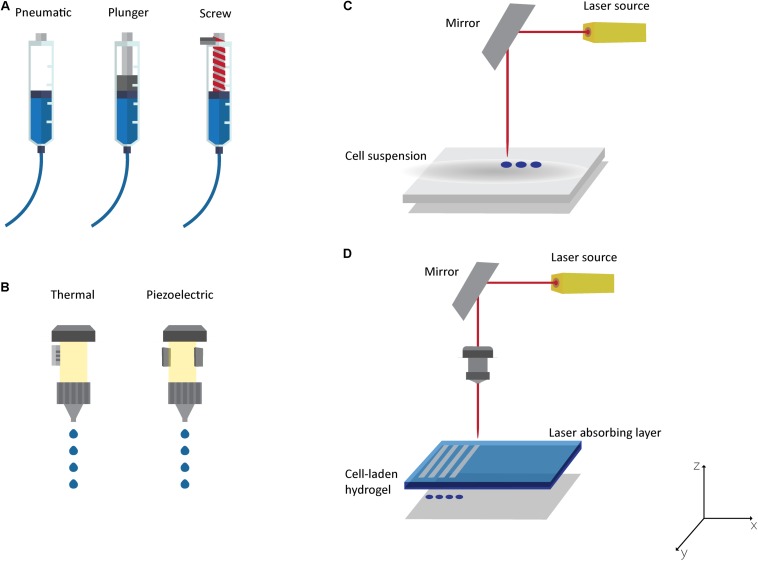
Bioprinting Technologies: **(A)** Microextrusion with pneumatic, plunger or screw-driven dispensing systems; **(B)** thermal or piezoelectric inkjet printing; laser-assisted bioprinting technologies: **(C)** laser guided direct cell printing and **(D)** laser-induced direct cell printing.

Three-dimensional (3D) printing emerged from additive manufacturing technology and has been mainly used to print biodegradable cell-free scaffolds in the field of TE. This top-down approach, in which cells are only seeded at the end of the printing process, presents some drawbacks, such as the inaccurate control over the distribution of cells, which results in a lack of structures to generate an appropriate ECM and the regeneration fails (Mandrycky et al., 2016). Consequently, 3D bioprinting emerged as one of the most promising bottom-up technologies for fabricating artificial tissues and organs (Murphy and Atala, 2014; Mandrycky et al., 2016), mainly due to its capacity to create complex structures, in a layer-by-layer fashion, with precise control over the deposition and positioning of biomaterials, growth factors, living cells, biochemical, and other functional components (Murphy and Atala, 2014; Seol et al., 2014). Traditionally, the bioprinting process is based on three individual and dependent phases. The first comprises medical imaging [computer tomography (CT) or magnetic resonance imaging (MRI)], which combined with computer-aided design/manufacturing (CAD/CAM) software, enables a 3D CAD model of a defect or an organ from the patient to be produced (Murphy and Atala, 2014; Seol et al., 2014; Bishop et al., 2017). From that, it is possible to create a code for the printer to generate a structure with an anatomical shape layer-by-layer (Seol et al., 2014; Mandrycky et al., 2016). The second phase corresponds to the printing and involves the selection of a bioprinting technique as well as the biomaterials for bioinks and scaffolds. The final properties of the structure will be affected by the selected techniques and materials. The last phase occurs when the structure is obtained and it can be: (i) maturated in a bioreactor before *in vivo* application, (ii) implanted as it is, or (iii) used as an *in vitro* model (Mandrycky et al., 2016; Bishop et al., 2017). Although all the phases are crucial in the bioprinting process, the printing stage is the most complex as it is crucial to obtain structures with the desired properties. Building human tissues by 3D bioprinting has received huge attention in the TE field due to its process flexibility and versatility. However, the need to implant the structure after production has generated several issues related to the integration with surrounding tissues, namely, (i) the difficulties to obtain a perfect fit for the geometry of the defect, (ii) the need for surgical debridement before scaffold implantation, and (iii) the contamination risk (Akilbekova and Mektepbayeva, 2017; Bella et al., 2018). To overcome these limitations, *in situ* bioprinting emerged to directly print biostructures into the patient’s affected tissue. In Campbell and Weiss (2007) proposed this concept for the first time, when they explored inkjet technology to *in situ* print fibrinogen, thrombin, and visualization dye into a rat calvarial defect. The authors stated at that time that they did not believe this approach could be adopted as a clinical methodology since it is not a simple off-the-shelf solution (Campbell and Weiss, 2007). *In situ* bioprinting has been adapted to the main bioprinting techniques to directly print into the defect, namely microextrusion, inkjet printing and laser-assisted printing (Figure 5). All of these techniques present different principles, characteristics, advantages, and disadvantages already described in detail elsewhere (Murphy and Atala, 2014; Seol et al., 2014; Mandrycky et al., 2016; Bishop et al., 2017; Peng et al., 2017; Jang et al., 2018). Briefly, the most common bioprinting technique is microextrusion (Figure 5A), which consists of a fluid-dispensing system that uses pneumatic pressure or mechanical forces (piston or screw) to print a continuous filament through a nozzle (Pati et al., 2015; Mandrycky et al., 2016; Ozbolat and Hospodiuk, 2016). Microextrusion allows the bioprinting of high viscosity bioinks (30 to >6 × 107 mPa/s), a wide selection of biomaterials, and very high cell densities (Murphy and Atala, 2014; Mandrycky et al., 2016). Other important advantages are the quality of the vertical structure and the ability to scale-up 3D structures (Seol et al., 2014; Mandrycky et al., 2016). The major disadvantages of this technique are related to low resolution (>100 μm) and low cell viability (40–80%) (Murphy and Atala, 2014; Seol et al., 2014).

The inkjet printing technique (Figure 5B) is defined as a non-contact bioprinting technique that generates bioink droplets (of picolitre volume) that are deposited onto a substrate by a thermal, piezoelectric, acoustic, or electromagnetic force (Seol et al., 2014; Zohora and Azim, 2014; Bishop et al., 2017). The major advantages of this technique are high print speed, high resolution (20–100 μm), multiple cell/material delivery, low cost, and good availability (Murphy and Atala, 2014; Mandrycky et al., 2016; Jang et al., 2018). Nevertheless, this technique presents some limitations related to poor printing fidelity, material viscosity (ideally < 10 mPa/s), cell density (< 106 cells/mL), frequent nozzle clogging, and process scale-up (Seol et al., 2014; Mandrycky et al., 2016; Bishop et al., 2017).

The laser-assisted bioprinting is an emerging technology that allows bioprinting cells and hydrogels with micron-scale resolution (Guillemot et al., 2010). The 3D structure can be obtained using a laser-guided direct cell printing, in which the laser traps and guides the cells in the desired direction (Figure 5C). On the other hand, the laser can induce direct cell printing in which two layers are needed: the energy-absorbing layer (containing the ribbon) on the top and a layer on the bottom containing the bioink (Figure 5D) (Devillard et al., 2014; Murphy and Atala, 2014; Mandrycky et al., 2016). During the printing process, the laser pulse is applied to stimulate an area on the absorbing layer of the ribbon to generate a high-pressure bubble that forces the bioink toward the collector (Guillemot et al., 2010; Bishop et al., 2017; Jang et al., 2018). For both approaches, after deposition, a crosslinking step is needed to maintain the desired architecture (Mandrycky et al., 2016; Jang et al., 2018). This bioprinting technique is a nozzle-free printing technique, and thus is clog-free (Jang et al., 2018). Other important advantages compared to the other bioprinting techniques are its ability to print moderately viscous materials (1–300 mPa/s), its high resolution (microscale), its increased cell viability (>95%), and its capacity to create multilayered cellular constructs (Murphy and Atala, 2014; Mandrycky et al., 2016; Jang et al., 2018; Kérourédan et al., 2018). Its major weaknesses are related to the side effects of cell exposure to the laser beam (currently not fully understood), and it being an expensive technique with complex control (Murphy and Atala, 2014; Mandrycky et al., 2016).

Additionally, stereolithography (SLA) is one of the most promising bioprinting techniques and uses light (UV, IR, or Vis) to photopolymerise a bioink according to a two-dimensional (2D) pattern, printing a 3D structure layer by layer (Ji and Guvendiren, 2017; Jang et al., 2018). Contrary to the previous techniques described, SLA does not dispense bioink. The bioink is placed into a reservoir with the platform moving in the z direction after each layer is photopolymerized (Mandrycky et al., 2016; Bishop et al., 2017). According to the technique configuration, there is no limitation in terms of bioink viscosity or structure geometry (Jang et al., 2018). Other important advantages are the high resolution (microscale) and high cell viability (>85%) (Mandrycky et al., 2016). However, it presents prolonged post-processing (Bishop et al., 2017) and is not suitable for *in situ* bioprinting due to its intrinsic characteristics, namely the need of a bioink reservoir.

Since the first approach in 2007, several research groups have explored *in situ* bioprinting using two different strategies: one based on accurate technique development to detect and print *in situ* (Cohen et al., 2010; Holzmond and Li, 2017; Li et al., 2017), and the other consists on *in situ* bioprinting in animal models to treat damaged tissues such as cartilage, bone, and skin (O’Connell et al., 2016; Duchi et al., 2017; Keriquel et al., 2017; Bella et al., 2018).

Until now, several studies have been conducted to achieve a proper *in situ* bioprinting (summarized in the Table 1), exploring and improving processes and bioinks, and evaluating the induced regeneration process. In Cohen et al. (2010), published a study describing for the first time how novel geometric feedback-based approaches and appropriate printing-material combinations allow the *in situ* repair of both chondral and osteochondral defects. The printing strategy was conducted by CT scanning of bone before and after creation of the defect to allow the resulting geometry to be traced back. Feature-based image registration was conducted to align the printing substrate within the printer (Cohen et al., 2010).

**TABLE 1:** Summary of *in situ* bioprinting studies.

***In situ* bioprinting technique**	**Target tissue/defect**	**Material**	**Cells or biomolecules**	**Substrate**	***In vitro***	***In vivo***	**Highlights**	**References**
Extrusion	Bone Osteochondral Chondral	Alginate Alginate Hyaluronic acid	n.a.	*Ex vivo* animal bone	n.a.	n.a.	Only feasibility of 3D printing and 3D scanning were evaluated	Li et al., 2017
Co-axial extrusion	Cartilage	Core: Gelatin methacryloyl/Hyaluronic acid methacrylate, GelMa/HAMa, Shell: GelMa/HAMa + lithium-acylphosphinate	Core: adipose-derived mesenchymal stem/stromal cells (ADSCs)	*In vivo* sheep model	Viability of the cells after processing	*In vivo* study demonstrated an early cartilage regeneration in sheep		Bella et al., 2018, O’Connell et al., 2016, Duchi et al., 2017, Hakimi et al., 2018
Laser-printing	Bone	Collagen/nano hydroxyapatite	D1 cell line (multipotent mouse bone marrow stromal precursor cells)	*In vivo* mouse calvarial defect model	Significant increase in metabolic activity from day 1 to 8, for ring and disk printing design, no differences between them	After 2 months, in the case of the disk printed geometry, the regeneration is homogeneous throughout the defect, in contrast to the ring geometry, where the regeneration is mainly observed at the periphery	Different cell printing geometries, showed different cellular arrangements that influence bone tissue regeneration	Keriquel et al., 2010; Keriquel et al., 2017
Laser-printing	Skin (full-thickness wounds)	Fibrin/collagen Thrombin	Amniotic fluid-derived stem cells (AFS) or bone marrow-derived cells (MSCs)	*In vivo* full-thickness skin wound nude mice model	Angiogenic growth factors secreted by AFS cells induce endothelial cells migration	AFS cells deposited in a collagen/fibrin gel accelerated closure of full-thickness wounds in mice compared to gel-only controls and was as effective as MSC treatments	Bioprinting provides the ability to deliver cells in a fast, off-the-shelf manner, facilitating quick wound coverage and closure, which is critical in cases of large full-thickness wounds or burns.	Skardal et al., 2012
Extrusion-based with microfluidic cartridge	Skin (full-thickness wounds)	Alginate, fibrin, collagen, or hyaluronic acid	Fibroblasts and keratinocytes	*In vivo* compatibility of *in situ* bioprinting in small animals (murine wound model) and large animals (porcine wound model) models.	> 90% viability	The selected bioinks did not inhibit granulation tissue formation or reepithelisation.	Single or multilayered sheets with different deposition configurations	Hakimi et al., 2018
Inkjet printing	Skin (full-thickness wounds)	Collagen, fibrinogen, thrombin	Fibroblasts and keratinocytes	*In vivo* compatibility of *in situ* bioprinting in small animals (murine wound model) and large animals (porcine wound model) models	n.a.	Printed skin cells were able to close the entire wound by 3 weeks post-surgery	Proof-of-concept of a mobile *in situ* skin bioprinting system with integrated imaging technology to provide rapid on-site management of full thickness wounds.	Albanna et al., 2019

Ideally, an *in situ* printing technique should scan the defect, identify the damaged area, and print new tissue accordingly. A study developed by Holzmond and Li (2017) presented a “certify-as-you-build” quality assurance system with the ability to monitor the printing process, detect the geometry using 3D digital image correlation (3D-DIC), and compare the printed geometry with the computer model to identify print errors *in situ*. A case study was developed using a fused filament fabrication (FFF) 3D printer and demonstrating the *in situ* error detection of local and global defects (Holzmond and Li, 2017).

Recently, Ding and Chang (2018) developed a novel *in situ* bioprinting workflow that integrates imaging of burn wounds with additive manufacturing to reduce the time of medical intervention and improving the efficacy of wound healing. The rapid production of patient-specific skin graft intend to eliminate the demand for the *in vitro* fabrication and culture of cell-laden tissue construct in a laboratory setting (Ding and Chang, 2018).

A study developed by Li et al. (2017) reported the combination of 3D scanning and 3D printing to treat bone and cartilage defects. Different defect models were created and *in situ* 3D bioprinting feasibility evaluated. High-resolution 3D scans were used to obtain the 3D digital models (defect and healthy part) and used Boolean operation to determine the shape of the defects and to import the target geometries to the 3D bioprinter. The results suggested that the correlating technology developed provides a novel methodology to treat open defects in the skeletal system and can be more effective in non-conventional cases (Li et al., 2017).

**FIGURE 6:**
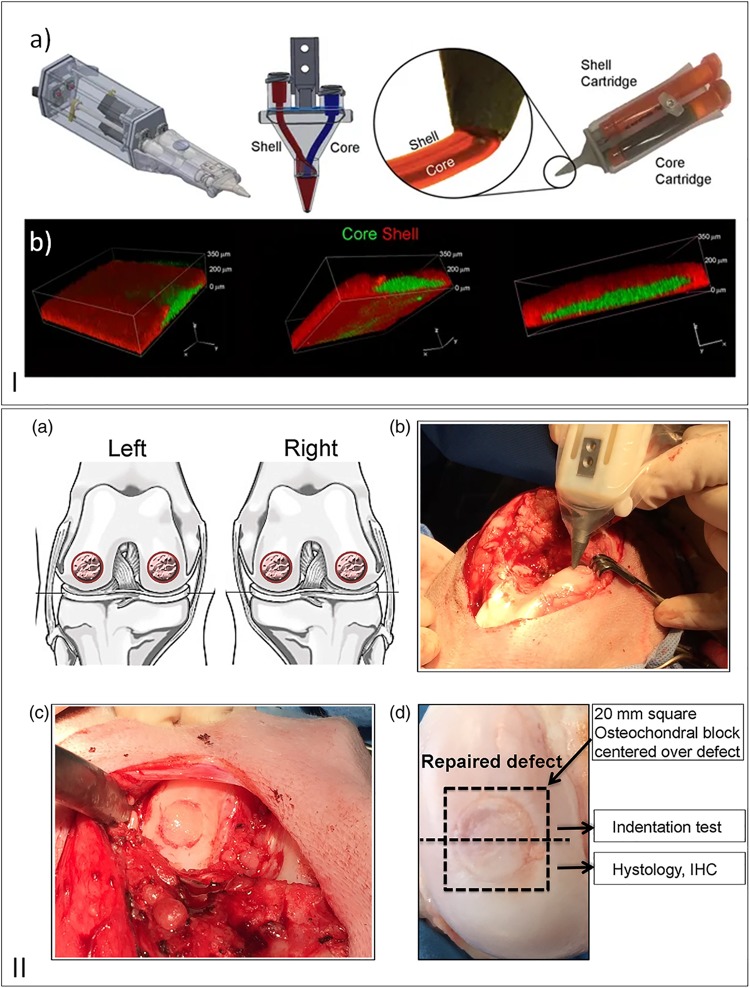
**(I)** (a) Schematic draw of 3D handheld printer by co-axial extrusion; (b) Representative 3D rendered confocal images of core/shell printed sample labeled with fluorescent beads (green: gelatin-methacryloyl/hyaluronic acid methacryloyl (GelMa/HAMa) plus 0.1% of lithium-acylphosphinate (LAP); red: GelMa/HAMa), reproduced with permission from Duchi et al. (2017). **(II)** (a) Schematic draw of full–thickness chondral defect made. (b) Intraoperative photographs of the Biopen in action. (c) Defect filled with hand–held *in situ* 3D printed bioscaffold using the biopen and coated with fibrin glue spray. (d) Macroscopic picture of the retrieved specimen, reproduced with permission from Bella et al. (2018).

Choong’s team (O’Connell et al., 2016) has been developing and improving an *in situ* handheld 3D bioprinting device (Biopen) for cartilage regeneration (Figure 6). The Biopen allows simultaneous coaxial deposition of living cells and biomaterials in a manual, direct−write manner. This device integrates bioink chambers, a multi-inlet extruder nozzle, a light source, and a motorized extrusion system (O’Connell et al., 2016; Duchi et al., 2017; Bella et al., 2018). *In vitro* experiments were performed verifying the viability of the cells after processing and an *in vivo* study demonstrated an earlier cartilage regeneration in sheep compared to the performance of bench-based printed bioscaffolds, microfractures, and the untreated groups (Duchi et al., 2017; Bella et al., 2018).

Some studies have been developed to achieve bone regeneration with *in situ* bioprinting. Keriquel et al. (2010) presented the first attempt to apply *in situ* bioprinting technologies *in vivo*. They designed a workstation dedicated to high-throughput biological laser printing and evaluated their system in a mouse calvarial defect model. Although the results revealed inconsistent reconstruction of the defects on test and control sites, the authors demonstrated that is possible to perform *in situ* and *in vivo* bioprinting (Keriquel et al., 2010). Recently, Keriquel et al. (2017) demonstrated that the laser-assisted bioprinting technique can be used in mice calvarial defect model to *in situ* bioprinting (mesenchymal stromal cells, collagen and nano-hydroxyapatite) to promote bone tissue regeneration. Furthermore, the cell bioprinting patterns directly influence the tissue regeneration (Keriquel et al., 2017).

Skin is the major organ in the human body and has several important functions (Casey, 2002; Reddy et al., 2013). Therefore, when damaged at full-thickness, the human life could be at risk (Yildirimer et al., 2012). Consequently, new and innovative strategies are required to reduce the time spent in hospital and to avoid conventional procedures, such as the use of autografts (Dias et al., 2016). Skardal et al. (2012) used laser-assisted bioprinting technology to treat full-thickness skin wounds in nu/nu mice. The study demonstrated that stem cells obtained from amniotic fluid and bioprinted in a fibrin/collagen hydrogel carrier are a promising approach for an effective wound healing therapy in clinical applications (Skardal et al., 2012). Recently, a hand-held skin bioprinter was developed by Hakimi et al. (2018), which is a compact extrusion-based approach that enables the *in situ* formation of biomaterials and skin tissue sheets of different architectures and compositions. Bioink solutions are spatially organized using a microfluidic cartridge and crosslinked immediately after deposition. According to the results, the authors demonstrated that the handheld bioprinter allows the bioprinting of striped, spotted, or fiber arrays sheets, and single and multilayered biomaterial sheets with different biopolymers (alginate, fibrin, collagen, and hyaluronic acid) and cell types (fibroblasts and keratinocytes). The *in vitro* and *in vivo* experiments demonstrated that skin architecture was mimicked and that it is possible to cover large wounded areas (Hakimi et al., 2018).

**FIGURE 7:**
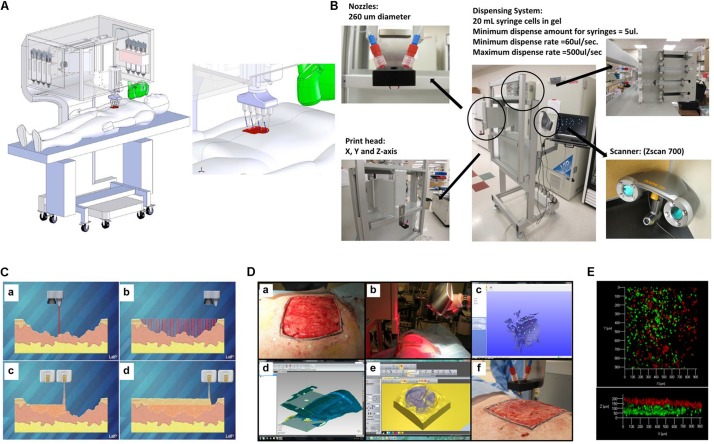
Skin bioprinter prototype and *in situ* bioprinting concept. **(A)** Schematic demonstrating scale, design, and components of the skin bioprinter. **(B)** The main components of the system consist of nozzles, driven by up to eight independently dispensing systems connected to a print-head with an XYZ movement system, in addition to the 3D wound scanner. **(C)** Skin bioprinting concept. **(D)** Example of skin bioprinting process, (a) markers were placed around the wound area used as reference points prior to scanning with a hand-held ZScanner Z700 scanner (b). (c–e) Geometric information obtained. (f) Printing accordingly the geometry. **(E)** Depositing of multiple cell types with high precision and control. Layering of fibroblasts (green) and keratinocytes (red) is shown, reproduced with permission from Albanna et al. (2019).

Recently, Albanna et al. (2019) study described a novel design and was a proof-of-concept validation of a portable skin bioprinting system that offers rapid on-site extensive wounds management (Figure 7). The system has an integrated imaging technology facilitating the precise delivery of cells (dermal fibroblasts and epidermal keratinocytes) into the damaged area. The cartridge-based delivery system used is similar to the conventional inkjet printing. According to the *in vivo* assays, excisional wounds showed rapid wound closure, reduced contraction and accelerated re-epithelialization when bioprinted with fibroblasts and keratinocytes in a hydrogel carrier. Moreover, histological results demonstrated that the new tissue is similar to healthy skin in terms of dermal structure and composition (Albanna et al., 2019).

However, this is an emerging field that requires more efforts to become a clinical reality. At the moment, the major restrictions are related to: (i) integrated systems that are needed to scan the damaged area and communicate with the printer to bioprint according to the defect area, (ii) bioprinting in non-horizontal surfaces, (iii) printers with high accuracy, (iv) bioinks with the ability to keep the structure instantly and to promote tissue regeneration, and (v) to guarantee that the *in situ* bioprinting does not affect, interfere with, or damage the surrounding tissues.

Additionally, despite the great potential of bioprinting and *in situ* bioprinting, some doubts have been raised regarding the processing effect on cell integrity and viability. Until now, few studies have been conducted to further understand cell/biomolecule damage and cell proliferative ability during and after printing. The study developed by Nishioka et al. (2004) described a direct correlation between the compression rate and peroxidase activity. Higher compression rates correlated with lower enzyme activity (Nishioka et al., 2004). Recently, Ning et al. (2018) studied the induced stress on the cells by bioprinting. Based on this, the team developed a novel method to characterize and qualify the cell damage caused by both shear and extensional stresses. The viability and proliferation ability of cells after bioprinting were investigated and the results illustrated that the process-induced forces affect not only cell viability but also their proliferative ability (Ning et al., 2018).

#### 4D *in situ* Bioprinting

The aforementioned 3D printing emerged to produce complex structures in several fields based on a layer-by-layer strategy. More recently, and due to its potential in the biomedical field, 3D bioprinting arose, which enables the production of complex structures with biological components (Mandrycky et al., 2016). However, one major limitation remains, since only the initial state of the printed structure is considered, and the influence of time and stimuli is ignored (Gao et al., 2016; Momeni et al., 2017; Morouço and Gil, 2019). Thus, 4D has been presented as the next generation of tissue regeneration and intends to mimic not only the organs’ or tissues’ architecture and properties, but also their dynamic function (Gao et al., 2016; Momeni et al., 2017). The 4D refers to stimulation and was described, for the first time, in 2013 by the Massachusetts Institute of Technology (MIT) (An et al., 2016; Yang et al., 2019). 4D structures are capable of self-assembly, multi-functionality, and self-repair, and are time-dependent, printer-independent, and predictable (Momeni et al., 2017). The concept of 4D emerged associated with biofabrication and, consequently, with bioprinting, and can be categorized into two main approaches, namely materials capable of deformation and structures that mature after printing (An et al., 2016; Gao et al., 2016). Materials capable of deformation can also be called responsive materials that are able to reshape or change their function according to external stimuli such as water, temperature, pH, light, and electrical or magnetic fields (Momeni et al., 2017; Ashammakhi et al., 2018; Betsch et al., 2018; Miri et al., 2018; Armstrong and Stevens, 2019).

**FIGURE 8:**
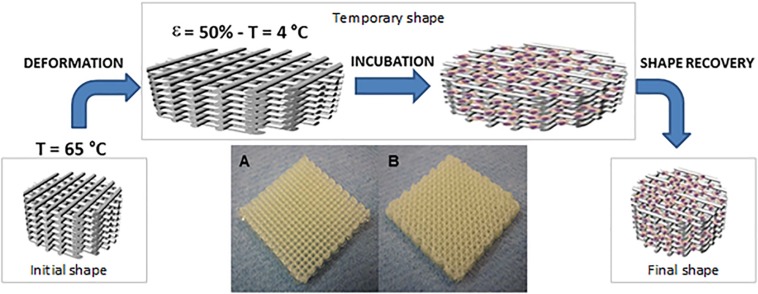
Schematic draw showing the working principles behind 4D scaffolds. By exploiting shape memory polymers (SMP), a polyurethane SMP additive manufactured scaffold is brought to 65°C where a temporary shape is imparted by applying 50% strain and fixed by cooling at 4°C. Scaffolds are then cultured at 30°C to allow cell adhesion and proliferation before restoring the temperature at 37°C and releasing the strain imparted to the scaffolds through the recovery of the permanent shape. 4D scaffolds in two different configurations have been tested: 0/90° **(A)** and 0/45° **(B)**. Scaffolds were produced with dimensions of 20 mm × 20 mm × 4 mm, reproduced with permission from Hendrikson et al. (2017).

There are several works exploring the stimuli-responsive materials in the biomedical field, such as Jamal et al. (2013), who demonstrated that a bilayered PEG hydrogel construct self-folded, after immersed in aqueous medium, into cylindrical structures of different radii with no adverse effect on the encapsulated cells. In addition, Hendrikson et al. (2017) described the printing of shape memory polyurethane (Figure 8), demonstrating that even when cells are seeded onto the scaffolds in the temporary shape, the permanent shape was recovered, thus fitting the requirements of a minimally invasive approach. Moreover, the cells were more elongated after shape recovery, indicating that a single mechanical stimulus induce changes in the adherent cells morphology (Hendrikson et al., 2017).

**FIGURE 9:**
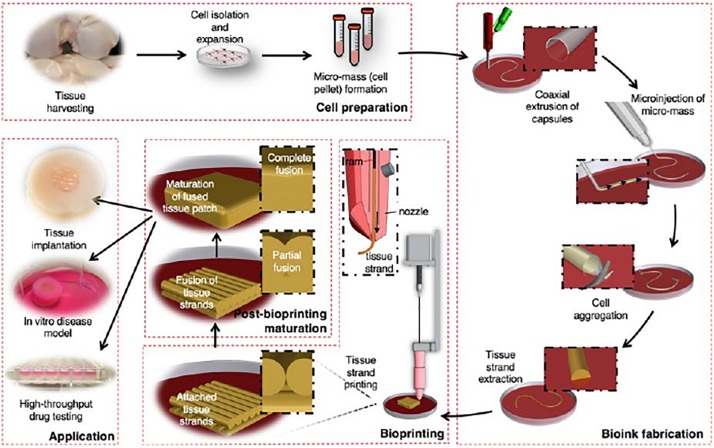
Schematic explaining the concept of tissue printing using tissue strands as a new bioink, reproduced with permission from Yu et al. (2016).

The structures’ maturation after bioprinting, considered as the second approach, is related to the tissue formation and maturation (Gao et al., 2016). Despite bioprinting allowing a precise control over the deposition of cells and/or biomolecules, the obtained structures cannot promote a complete tissue formation (An et al., 2016). Consequently, tissue or organ growth works as an incentive, stimulating the medical device to gradually break down and be absorbed by the body (An et al., 2016; Gao et al., 2016). However, scaffold-free strategies induce pattern changes over time due to the maturation, as a consequence of cell communication and self-organization (An et al., 2016). Yu et al. (2016) introduced a novel scalable bioink (scaffold-free), “tissue strands,” to facilitate the bioprinting of biomimetic tissues (Figure 9). The generated tissue strands are bioprintable, can be rapidly fused and maturated by self-assembly, can be bioprinted in solid form, do not need a liquid delivery medium during extrusion, and are no scaffold-dependent. According to the results, this approach can be a novel platform for tissue or organ biofabrication by taking advantage of the self-assembly ability of biological tissues, namely for articular cartilage tissue (Yu et al., 2016).

Until now, and according to An et al. (2016), “4D bioprinting is still more of a thing-to-be rather than a well-established matter of fact.” This approach, despite being in an early stage of development, can contribute significantly to the biomedical field by producing hierarchical and dynamic tissues. Moreover, this new generation of structures can be implemented not only by bioprinting processes, but also through other technologies. However, several issues remain unclear, such as the multiresponsive structures, how to achieve precise control over the deformation, and an *in vivo* evaluation. Despite the *in situ* bioprinting advantages and the potential advantages of *in situ* 4D, this approach has not been explored until now. Nevertheless, *in situ* 4D bioprinting will overcome the current limitation of 4D printing related to the adhesion between the structure and damaged tissue, in addition to achieving *in vitro* maturation prior to structure implantation.

#### Hybrid *in situ* Approach

*In situ* regeneration can revolutionize the future of clinical procedures, since with this new approach, the human body works like an “*in vivo* bioreactor,” providing the ideal environment for the regeneration of tissues (Wang et al., 2015; Ng et al., 2017). According to the strategies aforementioned, all technologies present some advantages and disadvantages in terms of their material range, accuracy, resolution, and target tissues. However, over the last few years, *in situ* bioprinting has gained notoriety due to the ability to print complex 3D bioengineering structures layer-by-layer with a high degree of flexibility and reproducibility (Chua and Yeong, 2014). Similarly to hybrid bioprinting, hybrid *in situ* bioprinting, through the combination of different printing strategies, is an interesting and attractive approach to fabricate *in vivo* 3D hierarchical structures combining in the same structure different scales from nano to macro (Kim et al., 2008; Xu et al., 2013). In fact, most of the *in situ* bioprinting techniques currently under study present the shape of the ECM analog deposited (droplets or continuous filaments) as their main difference, which is directly correlated with the type of material used, its viscosity, and the cell density allowed. Based on this, and to develop complex structures that mimic the ECM of the damaged tissue, it will be important to combine micro techniques, such as the conventional layer-by-layer techniques (inkjet printing, laser-assisted printing and microextrusion), with nanotechniques, such as electrospinning. Several studies have explored the combination of different *in vitro* bioprinting techniques and are described elsewhere (Canha-Gouveia et al., 2015; Dias et al., 2016). Currently, *in situ* bioprinting studies have demonstrated the potential of this approach, however, due to the *in vivo* conditions, many materials with high mechanical properties cannot be used as a result of the high temperatures required to print them. An alternative might be to integrate *in situ* spinning or *in situ* printing techniques, allowing the production of a complex and hierarchical structure able to mimic the biomechanical properties of the native ECM.

## Concluding Remarks and Future Prospects

Although the many successful *in situ* TE examples herein presented, they are not yet fully available to treat patients. Nevertheless, the existing technological toolbox for constructing tissue specific ECM analog biomaterials, directly at the defect site, has opened the possibility for more translational research in the upcoming years. For this to happen, the portability and fidelity of the proposed fabrication technologies need to be even more refined to meet the required simplicity of use in the surgical room in a relatively short period of time. Each *in situ* fabrication technology will have to be optimized to the type of biomaterial to be used and corresponding functional application. While in superficial wound lesions different approaches can be explored with a great degree of success, when looking at more complex and inaccessible tissues such as bone, cartilage or cardiovascular tissue, the size and shape of the defects requires specific biofabrication tools integrating the possibility of mapping the anatomy of the defect. Time is a very important parameter, since the prolonged fabrication associated with the scaling-up of large extensions of tissue might compromise its feasibility in the surgical room with consequences on cell viability, in case of a cell-laden approach. Therefore, more automated ways of loading and ejecting the cell−biomaterial suspensions are required in order to scale−up the bioconstructs fabrication.

It is also important to realize that complex biofabricated constructs require a high level of control and monitoring of biomaterials and, if appropriate, of the processed cells. The dynamics at the defect site include any physicochemical changes, such as dimensional stability (shrinking/swelling), degradation, and pH changes, which might occur in the biomaterials during the *in situ* fabrication and the following period. The tissue regeneration can be followed by assessing functional markers in real time, for example, by integrating biofabrication technologies with adequate sensors. As proposed by Holzmond and Li (2017), the concept of “certify-as-you-build” as a quality assurance system with the capability to monitor the fabrication process, should be extended to the post-processing stage to follow the events of tissue healing and regeneration and to ensure the expected therapeutic result.

Finally, it is obvious that the clinical application of *in situ* biopriting technologies will depend on fruitful collaborations across different disciplines, covering materials science and engineering, biochemistry, biology, and medicine, to foster not only progress in fundamental research but also translational research.

## Author Contributions

JD and AO designed the contents. JD wrote the *in situ* tissue regeneration, enabling approaches, hybrid *in situ* approaches, and 4D *in situ* bioprinting. NR wrote *in situ* spinning. SB-S wrote *in situ* gelling. AC-P wrote *in situ* spraying. AO wrote the sections “Introduction” and “Concluding Remarks and Future Prospects”. AO and NA provided the technical advice and critical revision of the manuscript. All authors provided critical feedback in the revision of the manuscript.

## Conflict of Interest

The authors declare that the research was conducted in the absence of any commercial or financial relationships that could be construed as a potential conflict of interest.
